# Exploring the trajectories of highly skilled migration law and policy in Japan and the UK

**DOI:** 10.1186/s40878-021-00251-3

**Published:** 2021-09-27

**Authors:** Daisuke Wakisaka, Paul James Cardwell

**Affiliations:** 1 Keidanren, 1-3-2, Otemachi, Chiyoda-ku, Tokyo, 100-8188 Japan; 2 University of Strathclyde, Law School, 141 St James Rd, Glasgow, G4 0LT UK

**Keywords:** Highly skilled migration, Migration policy, Migration law, Points-based system, Japan, United Kingdom, Brexit

## Abstract

Japan and the UK appear to have few commonalities in terms of their history of and approach to migration law and policy. However, strong similarities in their contemporary approaches can be detected. Migration sits at the very top of the national political agendas and both have undertaken successive, major policy reforms over the past decade. Both have governments publicly committed to policies to attract ‘highly skilled’ migrants, with a restrictive approach towards ‘unskilled’ migrants. This article draws out the similarities and differences of migration law and policy in Japan and the UK via their respective legislative structures and policy trajectories on highly skilled migration. The article argues that Japan and the UK promote a market-driven model which enables highly skilled migration to be ‘sold’ to publics believed to be hostile to increased migration. Yet, the rapid changes in policy and revising of applicable rules often prevents the successful recruitment of highly skilled migrants to both countries.

## Introduction

Japan and the UK have both made attracting highly skilled migrants (HSMs) a cornerstone of their respective migration policies. The length of their status as economic powerhouses, their different experiences of historical migration flows, and their recognition of (super)diversity in contemporary society differ. Yet, shared contemporary phenomena such as aging populations, colonial histories and national debates on how to resist or react to global patterns of migration have led both countries to undertake major reforms in recent years. Migration is a constant, salient political feature in both countries, and a heavy focus has been placed on policies to attract and support incoming HSMs, with a restrictive approach towards ‘unskilled’ migration.^1^ Both have underlined a ‘hostile environment’ for those in an irregular status (Kitagawa & Tanno, 2016; Goodfellow, 2019, also Sigona, Kato & Kuznetsova, this issue) and their resistance to asylum-seeking (see Phillimore et.al., this issue).

This article explores the respective trajectories which have led to the contemporary emphasis on HSM (highly skilled migration) as the cornerstone of migration policy in both Japan and the UK. The routes to this similar end-point differ, as does the success of policies and regulatory frameworks that both have put in place to meet their migration-related priorities. By tracing the dynamism of law and policy through three phases, we analyse the means by which both countries use migration law and policy to pursue broadly similar aims. After setting out how Japan and the UK can be seen in the global context of migration and national migration systems, the article examines the legislative frameworks and trajectories of policies on HSM in both countries to contour the longitudinal legal and policy framework for migration. The final part analyses our finding that the two countries promote a market-driven model which enables HSM to be ‘sold’ to publics who are perceived to be generally hostile to migration, though with limited success.

### Japan and the UK in the global migration context

The few case studies that compare Japan and the UK conclude that both have a restrictive approach in migration policies as ‘insular’ states (e.g. Phillimore, Liu-Farrer and Sigona, this issue; Strausz, 2019; Layton-Henry, 1992). Both are experiencing common challenges in terms of a shortage of skilled and unskilled labour, but resist (or attempt to resist) high(er) levels of immigration. Much of the recent discussion on UK immigration has focussed on the consequences of the UK’s withdrawal from the EU (‘Brexit’) and subsequent end to EU the free movement of workers under EU law. UK government discourse since 2016 has centred on the mantra of ‘taking back control’ of immigration law and policy, including on borders (see e.g. Donmez & Sutton, 2020; Slaven & Boswell, 2019). This discourse, inherited from the campaign to leave the EU, often rests on inaccurate characterisations of an unrestricted immigration policy as a consequence of EU membership and free movement principles. EU law does not cover migration from outside the EU and all Member States (including the UK) have used law and policy to regulate non-EU migration during the whole period of their memberships. In the post-Brexit regime, such tools are now applied to (potential) EU migrants to the UK too, which makes contemporary analysis and comparison of the UK and Japan pertinent. As both the UK and Japan witness a diversification of their societies (Phillimore, Liu-Farrer and Sigona, this issue), there is value in drawing out what the approaches to migration law and policy from two geographically distant, but major global economic players can tell us.

Long-standing restrictive migration policies in Japan and the emergence of the country as a 'migration state' (Hollifield & Sharpe, 2017) have attracted significant scholarly attention. Bartram (2000, 2005) contends that Japan in the 1960s and 70s represented a ‘negative case’ where the migrant ratio in the workforce had been considerably lower than the other industrial economies, while the economic growth had been fully achieved. He advocated the necessity of researching into the ‘negative case’ which the existing migration theories developed in the Western economies cannot explain.

Bartram’s (2000, 2005) argument has been principally developed in the area of unskilled migration in whiche guest workers have been accepted with much tighter restrictions, that is to say without settlement or family reunification rights. Other scholars have pointed to the culture of ‘developmental states’ as the key factor predicating the restrictive stances (Seol & Skrentny, 2009; Skrentny & Lee, 2014; Lee, 2018; Tian, 2019). The concept of ‘developmental states’ (coined by Johnson (1982)) originally instantiates the systematic and powerful bureaucracy in central governments playing the fundamental role in initiating industrial growth. The politics of developmental states also characterise immigration models in East Asia as those where migrants’ human rights are sacrificed in favour of economic growth. Seol and Skrentny (2009, p. 607) articulate that ‘Just as there is Asian model of development …there is an Asian model of immigration policy: low skilled migrants can work but they cannot bring family members and cannot, except in rare circumstances, settle’. Japan can be understood in this context, though the analysis below shows that it has shifted towards ‘skilled’ migration programmes that are increasingly common in global migration regimes (Cerna & Chou, 2014; Harvey, 2014).

Within the context of skilled migration debates, Japan and the UK serve as useful examples in comparing their respective policies. First, Castles et al., (2014) categorise Japan and the UK within the group of ‘immigration countries’. They differ from ‘traditional immigration countries’ (e.g., US, Canada, Australia, and New Zealand) where major proportions of contemporary populations were settled through large-scale immigration over several generations; and 'emigrant countries' (e.g., China, and many African countries). The characterisation of ‘immigration country’ implies those which tend to keep positive net migration flows over the long term. Immigration countries are likely to focus on labour, while family reunification sits closer to the centre of migration policies in ‘traditional immigration countries’.

Therefore, despite their geographical distance, contemporary Japan and the UK share a basic foundation on labour migration policies. Both are high-income economies and innovation leaders. Specifically, the countries share a focus on high-specification manufacturing rather than assembling. Hence, both emphasise the need for scientific specialists, which has been the building-block of their migration policies in 2010s (Koudo jinzai ukeire suishin kaigi, 2009; Migration Advisory Committee [MAC], 2010). As a result, Japan and the UK are also global competitors in attracting HSMs. As a related point, the Japan-UK bilateral relationship has long since been strong: attempts by the Thatcher government in the 1980s succeeded in convincing Japanese companies to invest in the UK (as a gateway to the EU single market), of which the best-known examples are the car factories (Suzuki, 2020). The strength of the Japan-UK bilateral relationship is manifested in the choice of migration policies. The Points-Based System (PBS) was introduced in Japan by referring to the UK equivalent (Koudo jinzai suishin kaigi, 2009). However, the recent UK–Japan Comprehensive Economic Partnership Agreement in 2020 does not substantially facilitate any form of migration between the two (Cardwell, 2020).

Until the present day, the major difference in migration law and policy between Japan and the UK had been the result of the UK’s membership of the EU. Free movement of workers between EU Member States is a core part of the Single Market, but the UK’s attempts before the referendum to ‘unpick’ this failed (Cardwell, 2016). Having left the EU, the UK Parliament passed the Immigration and Social Security Co-ordination (EU Withdrawal) Act 2020, which has extended the PBS to EU citizens. Therefore, in 2021 the UK finds itself in a more directly comparable situation to Japan in which HSMs are channelled through one main route, i.e. the PBS.

### Migration legislation in Japan and the UK

Japan and UK have complex and multi-layered migration law and regulatory frameworks that rely on primary, secondary/delegated, and quasi-legislation (see Table 1). The high place migration occupies on the political agenda tends to lead to frequent changes to (executive-led) secondary and quasi-legislation. This section provides a picture of the immigration legislation in Japan and the UK, subsequently showing how they share similar motivations in controlling migration and legislative outputs.

**Table 1:** legislative structure of immigration control in Japan and the UK

Types of legislation	Functions (examples)	Japan	UK
Primary Legislation	Prescribing basic systems	Immigration Act	Immigration Act 1971
Secondary Legislation	Clarifying definitions, Defining the overall policy directions	Cabinet Ordinances	Orders (Regulations)
	Clarifying conditions, procedures	Ministerial Ordinances	Rules Codes
Quasi Legislation	Further clarifying details, showing administrative understanding	Notifications	Government Circulars Operational Guidance

Source: Immigration Act (Japan), Immigration Act 1971 (UK), Immigration (Leave to Enter and Remain) Order 2000, Immigration Rules: Parts 1 to 14

#### Legislative frameworks

The legal architecture of migration in both Japan and the UK is built upon Immigration Acts as the core primary legislation. For Japan, this is the Immigration Control and Refugee Recognition Act (hereafter Immigration Act).^2^ The respective UK law is the Immigration Act 1971. UK immigration and citizenship law is not devolved to Scotland, Wales or Northern Ireland.^3^ Both Acts confer on the Minister of Justice (Japan) and the Home Secretary (UK) wide powers to make secondary legislation to prescribe conditions and procedures, and individual decisions on immigration. The structure of immigration legislation is thus hierarchical and, although there is no space here to analyse the large amount of secondary legislation, its importance to migration governance should not be underestimated. Similarly, the court systems in Japan and the UK play important roles in enforcing the law and, indirectly, holding Ministers and public bodies to account.

Both in Japan and the UK, immigrants must have a visa (‘entry clearance’ in the UK) to work.^4^ A visa, however, is just one condition to obtain a permit to enter and reside in the country (IOM, 2011). The Japanese Act establishes that an immigrant can reside lawfully only by being given a permit from the Immigration Authority. This is called ‘status of residence’. Its equivalent is ‘leave to enter’ (or ‘remain’) in the UK. The visa statistics may not however represent the whole population of documented immigrants residing in the countries: there may be individuals who obtained a visa but were refused entry on other grounds, or obtained a visa but did not migrate.^5^ Table 2 encapsulates the differences between visa and status of residence/leave to enter/remain.

**Table 2:** visa and entry process in Japan and the UK

	Step 1 Visa	Step 2 Immigration control	Step 3 Lawful status given
Japan	Obtain visa	Examined by an immigration officer at a port of entry	Given a **status of residence**
UK	Obtain visa (‘entry clearance’)		An entry clearance having effect as **leave to enter (remain)**

Source: Immigration Act (Japan), Immigration Act 1971 (UK)

**Table 3:** newly arrived HSMs in 2018^a^

Professor	3194
Artist	435
Religious Activities	872
Highly Skilled Professionals (i.e. through PBS)	531
Business manager	1790
Researcher	368
Instructor (e.g. language instructors)	3432
Engineer, Specialist in Humanities/ International Services	34,182
Intra-company transferee	9478
Skilled labour (e.g chefs)	3551
other HSMs	103
**total**	**57,936**

Source: Immigration Services Agency (2019)

^a^As UK statistics are only available in flow data shown in Table 4, we also cited the flow data here to compare the figures of both countries. We find that the stock data of HSMs represents the true picture of HSMs. Entertainers are not in the table as they tend to stay short-term (the number of residents is only 2389 in 2018)

**Table 4:** Newly arrived HSMs in UK (2018)

Tier 1	Tier 1: Entrepreneur	790
	General	215
	Investors	115
	Post-study	50
	Exceptional Talent.	165
Tier 2	General	25,000
	Intra-company Transfer	20,500
	Minister of Religion	695
	Sportsperson	1310
	**total**	**48,840**

Source: Home Office (2019)

**Table 5:** long-term migration inflows by type (thousand)

	year	work^a^	family	Humanitarian	others	free movement	International students^b^	trainees^c^
Japan	2018	66	31.9	0.1	17	NA	124.2	157.8
	2014	29.3	21.4	0.2	12.8	NA	82.5	98.7
	2010	19.3	21.9	0.4	14.1	NA	63.5	51.7
UK	2018	36.6	88.7	25.2	32.7	159.5	330.9	NA
	2014	79.2	64.4	17.2	22.4	128.2	missing	NA
	2010	84	64.7	21.3	22.3	98.3	234	NA

Source: OECD, (2012–2020)

^a^‘Work’ includes HSM schemes in both countries but encompasses other categories as well (e.g. Tier 5 in the UK); therefore, the numerical data is different from Tables 3 and 4

^b^ International students and trainees are temporary migrants in OECD figures

^c^ Trainees are mostly technical interns in Japan

 Table 2 represents the process of to how to gain residential status in the host country. This is only one part of the processes needed to settle. Japan and the UK do not offer a route to permanent residency at the first instance of their entry, unlike other countries such as Australia and Canada (sometimes referred to as ‘settler states’ (Dauvergne, 2016)). Both countries require further, stringent conditions for settlement, as we discuss later.

#### Legislation in Japan

In Japan, there are currently 29 classifications of status of residence which give foreign nationals legal grounds to reside for more than 3 months. The status of residence can be grouped into two types: activities or kinship. A status based on activities is restricted to those permitted by the Minister of Justice. Vetting of individuals and their sponsors is followed by the granting of permission only within the parameters of the activities allowed. These include studying and traineeships, but the interest here is on those who gain permission to work. There are three sub-groups within this category. First, foreign officials including diplomats. Second, and the focus here, HSMs (including business managers, legal or accounting services, medical services, researchers, instructors, engineers/specialists in humanities/international services, intra-company transferees, care specialists, entertainers and chefs). Third, ‘specified skilled workers’.^6^ Table 3 shows the number of HSMs entering Japan in 2018. The status of residence ‘Engineer, Specialist in Humanities/International Services’ is the most representative category of HSMs.

By contrast, residence based on kinship focusses on the relationship between the individual and Japan. This includes the spouse or child of Japanese citizens or permanent residents. It is therefore possible some of those exercising highly skilled professions gained their residency through this route, but they are not included in the statistics. Therefore, for the purposes of this article we are only focussing on the HSM route, whilst recognising the limitations of doing so.

To obtain a HSM visa, a job offer is indispensable, though no labour market test is required. The detailed conditions in defining ‘highly skilled’ depend on each status of residence, predicated on Ministerial Ordinances, two standards need to be satisfied: the equivalent of a graduate degree (or relevant 10-year professional career) and expected remuneration, comparable to a Japanese counterpart. Migrants can be accompanied by a spouse, but not family members such as parents.^7^ No language test is required although some jobs, including medical doctors, demand a prerequisite qualification in Japanese. Unlike the UK, no quota nor limit on length of stay applies. Individuals may apply for permanent residency after ten consecutive years of residence, except in a few cases. This requirement is stricter than other Asian high-income countries, such as the five-year minimum in South Korea and Taiwan.

#### Legislation in the UK

The central piece of immigration law in the UK is the Immigration Act 1971. The Act’s central concept is the ‘right of abode’ (section 1) for British citizens, and the subjecting of all those who do not fall into this category to the provisions of the Act (section 3). The Act is amended on an almost annual basis. Reflecting the political salience of immigration, the regularity of changes and the impact of case-law interpreting the provisions has resulted in an area of law of extreme complexity. Practitioner handbooks on UK Immigration Law (e.g. Phelan et al., 2018) run to over 2000 pages.

The other main source of immigration law in the UK is EU law. Until the UK left the EU on 31 January 2020, EU citizens were covered by the free movement rights guaranteed under the EU Treaties (Articles 26 and 45, Treaty on the Functioning of the EU (TFEU)). EU law has gradually evolved from a focus on workers to a more holistic approach for EU citizens and their family members (Shaw & Miller, 2013). EU law applied within the UK until the end of the transition period (31 December 2020), after which the provisions of UK immigration law now apply to EU citizens (see Peers, 2020). The Immigration and Social Security Coordination (EU Withdrawal) Act 2020 ended free movement rights and subjects EU citizens to the same regime as for non-EU citizens.

A long-term migrant resident obtains the leave to enter/remain, which from 2021 includes EU citizens. The PBS, put in place by the Labour government in 2008, was set out in ‘tiers’, which replaced a previous system of work permits. There have been numerous changes since then, to the extent that the PBS is ‘difficult to navigate successfully’ (Clayton & Firth, 2018). Tiers consisted of: 1 (entrepreneur, investor or ‘exceptional talent’); 2 (skilled); 3 (unskilled); 4 (students) and 5 (temporary/youth mobility, e.g. working holidays). Tier 3 was never used since migration from the EU, especially after the EU enlargements in 2004 to countries in Central and Eastern Europe, largely fulfilled domestic need. Tier 1 was originally to be used by HSMs without a job offer or sponsor, but this option was closed by the Conservative-Liberal Democrat government in 2010 and gradually replaced by more specific sub-categories (discussed below) (Gower, 2015).

Therefore, the UK equivalent of HSMs in Japan is primarily Tier 2. Like Japan, it is possible that HSMs enter the UK through other routes, and so it is important to note that the data will not necessarily capture everyone with the same levels of skills.

To obtain a Tier 2 visa, an applicant must hold a job offer from an employer. Tier 2 visas are intended to be used only when a vacancy cannot be filled by a UK worker, therefore, a labour market test is required unless the job appears on a list of ‘shortage’ occupations which is updated periodically. The applicant must be ‘skilled’, defined as being at graduate level, and at the time of writing, be paid above £30,000 per year (£20,800 for entrants under 26), pass an English test and pay the fee (between £232 and £1220). Fees are reduced where there is a shortage of workers according to a published list, often including engineers, doctors, nurses and architects. A Tier 2 visa holder is only entitled to remain in the UK for up to 5 years. To reside longer, they are required to gain permanent residency (‘indefinite leave to remain’) with conditions including a citizenship test and an annual salary threshold of £36,200 (in 2020). Only 6% of Tier 2 migrants entering in 2013 gained permanent residency (Migration Advisory Committee [MAC], 2020). Table 4 shows the figure of HSMs entering UK in 2018; Tier 2 General and intra-company transfer are the two major routes of HSMs.

### The trajectory of migration policies in Japan and the UK

HSM policies have been developed as part of a global ‘war for talent’ which began to appear in the 1990s (Beechler & Woodward, 2009; Chambers et al., 1998). In this section, we break down the development into three phases, which are predicated on the respective societal, economic and political backgrounds in Japan and the UK. In so doing, we place the HSM changes in the wider context of migration debates (e.g. unskilled migration for Japan and EU free movement for the UK).

#### Japan’s three phases in skilled migration policies

The Japanese government started to proactively seek HSMs from 1988. We term this the beginning of phase 1 (1988–2000). Until the late 1980s, Japan’s official stance was that it needed no migration, as the existing workforce covered labour demand (Kondo, 2002; Mori, 1997). The late 1980s saw a rapid economic globalisation, an inflated bubble economy and labour shortages. The government drastically changed its policy orientation and endorsed the third Basic Employment Measures Plan in June 1988. This plan adopted a new policy to admit HSMs whilst restricting unskilled migration:

Japan accepts foreign labour of the expert/technical sectors more proactively than ever to revitalise the socio-economy and promote internationalisation…Regarding unskilled labour…it is essential to cope with the issue with thorough deliberation based on a consensus among the Japanese people. (Japanese Government, 1988)

This decision has remained unchanged, with its core principle defining Japan’s long-term official stance (e.g., Ministry of Justice (MOJ), 2015b). Nevertheless, this official position has not prevented the government from increasing the number of unskilled migrants as guest workers through various mechanisms, such as the Technical Training Internship Programme, to fill labour shortages (Roberts, 2017; Surak, 2018). Given the fundamental change in direction in 1988, the Immigration Act was drastically amended in 1989 and expanded the categories of status of residence for HSMs, for example by creating a new status for intra-company transferees and medical professionals. This amendment also introduced a new status of ‘long-term resident’, primarily for *Nikkeijin* (overseas citizens of Japanese descent) to allow them to work with fewer restrictions. The majority of literature focuses on this *Nikkeijin* policy change with regard to 1989 reform (e.g. Cornelius & Tsuda, 2004; Tian, 2019), but it was also an important turning point for HSM policy.

Phase 2 (2000–2012) began to respond to Japan’s social and industrial changes. After the collapse of the bubble economy in the 1990s, the government realised the necessity of upgrading its industrial structure to higher-value, IT-driven models based on global supply chains. The Ministry of Justice [MOJ], (2000) declared in its 2nd Basic Plan for Immigration Control the ‘smooth acceptance of foreign nationals in response to new domestic and international social needs’, pointing to the necessity to develop IT industries. For this purpose, the government gradually relaxed the conditions and facilitated the administrative processes for HSMs. For example, adopting the mutual recognition of IT engineering qualifications with Asian economies^8^ enabled Japanese companies to hire HSMs with no tertiary degree. As HSMs had been defined as being at graduate level, this policy change brought about a novel approach in identifying skills. Employers also enjoyed a measure to shorten the examining process, as the MOJ declared the aim for 2 weeks (now 10 days) for a decision if a worker is hired by a company listed on the Japanese stock exchange or equivalent (Ministry of Justice [MOJ], 2015a; Wakisaka, 2018).

Japan’s population started to decline in 2008, prompting the government to consider migration as a means to sustain the economy against the background of one of the world’s lowest birth rates. However, the government was not eager to change the fundamental rule to only accept HSMs (Immigration Services Agency, 2019) and challenge the framing of increased migration as an issue of national and economic security (Vogt, 2014). The policy priority has remained focussed on attracting HSMs rather than unskilled migrants to increase the working-age population in Japan. *Keidanren* (Japan Business Federation), representing Japan’s large enterprises, has constantly advocated for increasing the number of HSMs, while remaining relatively low-key on unskilled migration (Keidanren, 2008). There is evidence that local governments advocated for more migration-friendly policies to stem regional population decline (Milly, 2014).

In 2009, the government’s Council for the Promotion of Acceptance of Highly Skilled Professionals produced a ground-breaking proposal to boost the number of HSMs by introducing the ‘Points-Based Preferential Treatment for Highly Skilled Foreign Professionals’ (PBS). Council members included representatives from labour unions, who were strongly against accepting unskilled migrants (see, for example, the meeting minutes of Kodo jinzai ukeire suishin kaigi, 2009). The PBS was finally implemented in 2012.

The PBS was the Japanese government’s first proactive policy to attract and retain HSMs. The formal name of the system is ‘Points-Based Preferential Immigration Treatment for Highly Skilled Foreign Professionals’. As the name indicates, it provides migrants approved to be ‘Highly Skilled Foreign Professionals’ with preferential treatment, such as fast-track permanent residency, not granted to other migrants.

Phase 2 is an important period in that the government changed its policies to treat HSMs as potential immigrants rather than guest workers; therefore, not only entry but also settlement policies were discussed and developed from this period (Ministry of Justice [MOJ], 2000). However, these reforms remained rather cosmetic, leading to technical revisions without amending laws. In other words, the government mainly relied on the reforms of secondary and quasi-legislation. This may be attributed to the fact that the Japanese political system was unstable in the latter half of the phase with a hung parliament from 2007 and change of ruling party from 2009. It can be argued that the legislative reforms were not possible because of the political turmoil, subsequently the overall reforms stayed low-key (Musashi, 2013). Nevertheless, more importantly, there was no drastic policy change even though the ruling party changed from the Liberal Democratic Party (LDP) (right-leaning) to Democratic Party (left-leaning) in 2009 when the hung parliament was temporarily resolved with a coalition of the Democratic Party and the Social Democratic Party being in the majority. This suggests a political change did not necessarily influence migration policies in Japan.

Phase 3 (2013-present) represents a period of more economically-driven migration policy-making. The government situated HSMs centrally in its economic policy. Former LDP Prime Minister Shinzo Abe returned to power in 2012 and embarked on a series of economic reforms (often known as ‘Abenomics’).^9^ His reforms included upgrading the PBS, as it fell behind the expectations (Oishi, 2014). The number of migrants through the PBS route was only 434 for the initial 11 months, accounting for 0.2% of the total number of HSMs. The government acknowledged the faults of the initial scheme (Immigration Services Agency, 2019). Measures included lowering the salary threshold and giving more generous exclusive packages such as fast-track permanent residency in only 1 year. Thanks to this upgrade, approval figures skyrocketed from 579 in June 2013 to 18,286 in June 2019 (Immigration Services Agency, 2019). The goal to increase to 10,000 at the end of 2020 (Japanese government, 2017) was achieved 3 years early.

Parallel to the PBS reforms, Parliament amended the Immigration Act in 2014 to streamline the categories of status of residence, which allows HSMs to have more flexible choice in choosing their career in Japan. In the former systems, the status of residence of an ‘engineer’ (for those with a scientific degree) and ‘specialist in humanities/international services’ (for those with a humanities/social sciences/law degree) were clearly separated. However, the university degree did not necessarily represent actual careers. For example, an engineer graduate may work as an investment banker which was generally deemed as a ‘specialist in humanities/international services’. This inappropriate categorization sometimes discouraged migrants from pursuing a different career from their graduate degrees.

Overall, the reforms in the phase 3 are characterized by two points. First, active ‘attracting’ of HSMs rather than passive ‘receiving’ was brought to the fore in the policies. Amid the global race for talent, the government gradually recognised that Japan is  in competition with other states to attract HSMs (Prime Minister’s Office, 2020). Second, these reforms are more drastic than the previous phase, requiring the amendment of laws rather than minor revisions. In summary, Phase 3 is the period when Japan recognised the limitations of its approach and made efforts, both in terms of reforming the legislation and changing the basis of the policy, to attract HSMs via more attractive and flexible packages. In this respect, the government’s claim of success of the changes (Immigration Services Agency, 2019) appears to demonstrate that Japan was serious about increasing the take-up of its PBS route, and that changes were not merely cosmetic.

#### UK’s three phases in skilled migration policies

Unlike Japan, the UK proactively encouraged migration from its (former) colonies after World War II to fill labour shortages. But a backlash against migration became a growing political issue from the 1960s and sparked a move to reduce and restrict migration routes (Layton-Henry, 1992). The post-war industrial context meant that attracting HSMs was not an express policy but one which emerged gradually over time. For the purposes of analysis here, for the UK the decade from 1997 to 2007 is Phase 1. The phase started when the Labour government of Tony Blair came into power and proactively reformed the policies to recruit HSMs from outside the EU. The Highly Skilled Migration Programme (HSMP) was introduced in 2002.

The HSMP was based on a points system allocated to attributions including educational record, work experiences and earnings. With sufficient points, s/he was given permission to enter without a job offer for one year. The permit was renewable three times, following which an application for permanent residency was possible. The requirement was to take active steps to be ‘economically active’ and thus there was no need to retain a highly-skilled job. In 2006, the programme was revised to require fulfilling the points in renewing the status. The points threshold was lifted from 65 to 75 points. The government initially aimed to increase the number of medical doctors (via additional points) and MBA degree holders, but these targeted groups comprised only 2.8% of all the successful applicants (Migration Advisory Committee [MAC], 2020).

Although the HSMP failed to deliver satisfactory outcomes by attracting HSMs in sufficient quantities (Home Office, 2007), the government continued policies to attract ‘the best and brightest’. During the 2005 General Election campaign, Prime Minister Tony Blair proposed ‘the type of points system used in Australia, for example, to help ensure our economy gets the skills we need’ (Blair, 2005) even though a PBS was already in place. As a result, the government introduced greater selectivity, and streamlined the 80+, complex immigration categories (Home Office, 2006). At this point, UK migration policies entered into a brief new phase.

The newly-endorsed PBS represents the short-lived Phase 2 (2008–2010)*.* The HSMP was replaced by a ‘tier’ system. Tiers 1 and 2 applied to those without and with a job offer respectively.^10^ Tier 1 required stricter conditions and higher number of points to be admitted and was expected to ‘widen the pool of highly skilled individuals and maintain labour market flexibility’ (Migration Advisory Committee [MAC], 2020, p. 44). Unlike the HSMP, conditions such as English language proficiency were required. The points system became stricter in 2009 to make it even more selective, but phase 2 was cut short due to the change in government to a Conservative-Liberal Democrat coalition, which turned the PBS from an ‘admissionist’ to a ‘restrictionist’ mechanism as the new government turned its attention to reducing overall net migration.

During Phase 3 (2010-present)*,* the aim of PBS of attracting global talent was still present in political discourse about the value of migration to the UK. However, ever further limitations and conditions were placed. As Prime Minister (2010–2016), David Cameron went beyond merely trying to be selective, with a commitment at the heart of his political messaging to reduce net migration from ‘hundreds of thousands’ to ‘tens of thousands’ (Cameron, 2011). Since the UK was not able to control EU free movement, the target to reduce the net migration was thus primarily focused on non-EU migrants via work, study and family routes. Government policy referred to limiting the number of ‘non-EU economic migrants’, without specifically mentioning HSMs (UK Government, 2010).

Thus, in 2010, the government tightened the conditions of the PBS, setting the minimum-required earnings to £25,000 p.a. and limiting the applicants to the post-graduate degree for Tier 1.^11^ The points requirement was further raised (95 to 100). A maximum monthly quota of 600 visas was introduced and the government closed the new initial application of Tier 1 in April 2011. It was instead replaced by Tier 1 (‘exceptional talent’) in August 2011 but with much tighter restrictions to prove exceptional talents. Tier 1 (exceptional talent) had an annual cap of 1000 which was doubled to 2000 in 2017 although it has never reached the limit. The largest annual number of successful applicants was approximately 600 in 2018. According to the MAC, an independent, public body that advises the government on migration, the low success rate was due to the strict conditions and procedures (Migration Advisory Committee [MAC], 2020). At the same time, the UK government, along with others in the EU but unlike Japan, facilitates a route to residence via large investments (Dzankic, 2015; Parker, 2017). This has not been without accusations of allowing money stemming from corruption into the UK (Transparency International, 2015).

Public opinion on immigration in recent years in the UK has shifted, insofar as it was a headline issue from 2001 to 2016 and has since declined but remains salient (Blinder & Richards, 2020). Public opinion remains more favourable to HSM, with recent analysis showing that the British population attaches higher importance to skills than other factors, such as skin colour or religion (Blinder & Richards, 2020; Heath & Richards, 2018). The Conservative government elected in December 2019 made an explicit commitment to end EU free movement. As such, the Immigration and Social Security Co-ordination (EU Withdrawal) Act 2020 has placed potential HSMs from the EU under the same non-EU regime (UK Government, 2020). This effectively means that the UK has closed one of the major, straightforward HSM routes and imposed greater formalities on citizens from its neighbours (except Ireland) as well as outgoing UK migrants to the EU. The extension of the PBS to EU citizens is likely to herald a major shift in UK migration patterns and one which might reveal that the much-lauded PBS reflects emotionally-based, rather than economically-based, policy-making (Cardwell & Da Lomba, 2020). If this is the case, particularly if there is a decline in HSMs in the UK (caused by EU HSMs leaving) then we might expect the HSM regime to become more flexible in response. This, however, is not guaranteed, as the lack of success and constant changes to the PBS thus far suggests.

### Policy outcomes and challenges in Japan and the UK

Japan and the UK appear to have arrived at a similar point: both have pursued successive policies changes with a view to attracting HSMs. And yet, the frequent changes to the legal provisions and categorisations, shows that turning a stated policy objective into reality is more difficult. Recent success in Japan in increasing HSMs can be contrasted with the UK, where political leaders ostensibly flown the banner for attracting the ‘brightest and the best’, but have consistently tightened PBS restrictions. The post-Brexit extension of the PBS to EU citizens will test whether potential HSMs from nearby countries will be attracted to the UK.

Table 5 depicts the recent inflows of migrants to Japan and the UK sums up our findings, using OECD Migration Outlook data which attempts to standardise the statistics across states.^12^ The inflow of ‘work’ migrants into Japan has tripled from 2010 (Phase 2) to 2018 (Phase 3), whereas that of UK has more than halved. Although the UK government tightened the entry of HSMs through the PBS scheme as discussed, this policy may have instead increased the number of EU migrants via free movement (over 50% increase from 2014 to 2018) as their substitutes (Rienzo & Vargas-Silva, 2014). This indicated that despite its rhetoric, the UK government could not ‘control’ migration in the way its official stance suggested.

Meanwhile, the statistics suggest that Japan has been successful in attracting HSMs. However, the overall picture of migration inflow offers other implications. The number of trainees (i.e. technical interns) has risen steeply, far outnumbering HSMs. Despite the prioritisation of HSMs, the data indicates trainees as less-skilled migrants were in fact much more successful in migrating to Japan. In sum, the policies on HSMs are mixed in terms of their success despite the political emphasis placed on this migration route.

Relating to the skill arguments, both countries remain in a transitional period of major policy shifts. As already shown, EU citizens must now use the PBS route, but its outcomes are unclear. From 2019 to 2020, the UK government expanded the Shortage Occupation List, encompassing more migrants in healthcare and IT sectors to respond to the labour shortages, in addition to abolishing market tests and quotas in the occupations listed. These reforms respond to employers’ needs who have struggled to secure sufficient labour (Migration Advisory Committee [MAC], 2020).

In 2019, labour shortages prompted Japan to launch a new programme called ‘specified skilled workers (SSW)’ which ‘stretched the bottom tier of the ‘skill’ category’ (Oishi, 2020, p. 7). SSW has two sub-categories: SSW1 (as equivalent to graduate technical interns, i.e., not HSMs but with three-year professional experiences compared to 10-years); and, SSW2 (as equivalent to HSMs with eligibility to bring family members and gain permanent residency on the same conditions).^13^ While SSW1 has 14 sectors to work (e.g. care, food service industries), SSW2 has only 2 (construction and shipbuilding/ship machinery). Although the new programme was much anticipated by stakeholders including industries and local communities suffering from acute labour shortages, the number of SSW1 as of 2020 was 15,663, comprising only 4.5% of the target for the initial 5 years (Immigration Services Agency, 2020). No SSW2 were granted since its screening criteria and application processes remain undefined after 2 years of implementation.

The shifts in Japan and UK reveal the challenges of ‘skills’ arguments in that they are closely related to labour shortages; it is unclear, once the labour shortages are alleviated, how migrants through the new schemes become non-HSMs. For example, SSW is the first ‘skills’ programme in Japan defined by labour shortages, but the COVID-19 pandemic has suddenly relieved the shortages in the hospitality industries. As a result, there are only 67 SSW1 migrants in the hotel industry (initial target: 22,000) and 998 in food service industries (target: 53,000) in 2020. It is quite telling that policy-makers are in fact more interested in *labour* rather than *skills* shortages, while ostensibly emphasizing the latter.

Another important lesson from the recent reforms is that the definition of ‘skills’ can be conveniently defined and fluid. Both Japan and UK have traditionally defined HSMs as either those with a university degree or sufficient professional experience, but the parameters are flexibly tuned in line with various needs, particularly labour demands. The fact is that ‘skills’ is context dependent and fluidly (un)created by the market as there is no universal definition of 'skills'. Rather, the use of 'skills' is the product of social and political construction (Kofman & Raghuram, 2015; Oishi, 2020). Our analysis calls for more scrutiny on ever-increasing ‘skills’ debates without taking policy reforms at face value.

## Conclusion

Comparing Japan and the UK in terms of migration is undoubtedly marked by more generalised conclusions about the approaches to migration in the round and diversity. In respect of the latter, there are marked differences between the two countries: whereas cultural and ethnic diversity has long been recognised as an integral part of the make-up of the UK, Japan is only making the first steps on this path (see Igarashi and Laurence, this issue).

If some of the historical and cultural context for their respective approaches to migration is put to one side, the law and policy on HSM can be seen to be remarkably similar. Japan and the UK’s approach to HSM is, in official discourse, reliant on the positive economic effects of HSM and fulfilling particular needs that cannot be met adequately within the countries. Both countries present themselves as welcoming to those with skills, whilst appearing to downplay any negative popular reaction to increasing migration. This is done by suggesting that HSMs are, by their very nature, a self-selecting small group and that they will be educated, high-earning and able to integrate into society. This in turn makes the immigration argument easier to ‘sell’ to populations who are believed to be sceptical or hostile to any actions that would seem to lead to an increase in migration. The discourse on borders and controlling are powerful in the context of both Britain and Japan being island nations, a readily visible feature of discourse in both countries.

Attempting to achieve the aims of attracting HSMs via frequent law and policy changes reveals strong similarities between Japan and the UK. In both, the core legal architecture remains rather stable, but the secondary legislation is subject to many changes. In both the UK and Japan, over the past 15 years changes have been made by newly elected governments soon after taking power: this appears to reflect the political saliency of migration rather than a need (economic or otherwise) to make the immigration system ‘work’ for migrants or potential migrants. Indeed, in both Japan and the UK, migration policy appears to follow a trajectory of trying to attract potential HSMs whilst also resisting (overt) liberalisation.

In neither country have the explicit aims on HSM been expressly fulfilled. This brings the argument (almost) full circle: if the policies put in place were left to develop over time, rather than being subject to more rapid changes by governments keen to show that they are ‘doing’ something, then a longer-term perspective on how migration could be better governed would be possible. The legal structures surrounding migration are, in both countries, flexible and highly reliant on the powers of the respective governments to change as they see fit. The end of free movement in the UK is the most recent and significant change likely to have an impact on migration to (and from) the UK. This measure has been promoted as a headline UK government commitment to ‘take back control’ of immigration and borders after Brexit. Governments are thus able to respond to the saliency of migration in public debates by promising to, and subsequently enacting, changes including ‘tightening up’ of the systems. The problem in doing so is that any gains might be short-term: the evidence in this article is that both countries have launched initiatives on HSM only to change them soon after. Since using HSM is a means to confront longer-term problems such as population decline and ensuring economic competitiveness, then frequent changes and ever-increasingly complexity seem less likely to meet these aims than those for domestic (political) consumption.

Thus, Japan and the UK appear to have arrived at the same point. Both are in need of attracting HSMs but unclear whether the law and policies will meet their aims. The respective policies appear to fall within the scope of a market-driven approach: that both provide attractive routes to HSMs in countries across the world who might consider pursuing such opportunities. Yet, there is an important caveat. A market-driven approach suggests an openness in the rhetoric about attracting HSMs, matched with a regulatory regime underlining its simplicity, speed and efficiency. Our analysis above shows that this is far from the case. Furthermore, given the emphasis on restricting other routes to migration, it may be that both countries are preventing themselves from confronting longer-term societal problems by pursuing shorter-term, political gains.

Since there are a few signs that either Japan or the UK will embark on a wider-scale liberalisation of migration policy, future research on HSM could investigate the extent to which geography, cultural or other factors affect the choices made by (potential) HSMs and uncover the links between, for example, attracting HSMs and citizenship. In this respect, there remain strong differences between the UK and Japan, in particular since the latter does not allow dual citizenship. Thus, to do so would necessitate a clearer understanding of the drivers, but also the changing patterns of where potential HSMs are located across the globe. In this respect, research on HSM has much to inform future law and policy-making in both Japan and the UK and whether attracting HSMs could be more effective is necessary, whilst bearing in mind the continued politicised context of migration law and policy. At the same time, the success of attracting HSMs for both Japan and the UK depends on how both countries fare in the global ‘war for talent’.

## Data Availability

All data generated and analysed during this study are clarified in this published article where possible.

## Abbreviations

HSM: Highly skilled migration
HSMs: Highly skilled migrants
HSMP: Highly Skilled Migration Programme (UK)
LDP: Liberal Democratic Party (Japan)
MAC: Migration Advisory Committee (UK)
MOJ: Ministry of Justice (Japan)
PBS: Points-based system
SSW: Specified skilled workers (Japan)

## Footnotes

1 We do not regard ‘unskilled’ as an appropriate characterisation of migrants who falls outside the ‘highly skilled’ category. We use the term here because it is the official term used by both the UK and Japanese governments.

2 This article follows the practice of not labelling the established year with the law when referring to Japanese legislation (Sato, 2017; Sellek, 2001). However, the Act was first passed in 1951.

3 See, for example, Scotland Act 1998, Schedule 5, Section B6.

4 Except for EU/EEA citizens (before 31 December 2020) or Irish citizens.

5 Japan issues statistical data on both visa and status of residence although the UK does not.

6 The ‘specified skilled workers’ category was launched in 2018 and is discussed below.

7 Except for the category of ‘Highly Skilled Professionals’ who are granted to bring their parents and domestic workers on certain conditions.

8 These economies were identified as Singapore, South Korea, China, the Philippines, Vietnam, Myanmar and Taiwan from 2001 to 2004.

9 ‘Abenomics’ are comprised of (1) robust monetary policies by the Central Bank, (2) the large-scale and flexible public expenditure, and (3) growth strategies into which migration policies are incorporated.

10 Tier 1 had five categories: (1) General, (2) Entrepreneur, (3) Investor, (4) Persons of Exceptional Talent, (5) Post-study work. Each of them required different conditions and no job offer. Unless specified, this article describes Tier 1 (General)/Tier 2 (General) as they were the centrepieces of the skilled migration programmes.

11 The post-graduate degree requirement was lifted in the following year.

12 While Japan’s statistics are complete survey from the immigration control, UK adopts sampling survey which is less reliable. EU free movement makes it more difficult to grasp UK’s migration data precisely. Therefore, we used OECD data which was, to the extent possible, harmonised to make a comparative analysis.

13 SSW 2 should have ‘proficient skills’ while SSW 1’s skills ‘require considerable knowledge or experience’ (Immigration Services Agency, 2019).
